# Clinicohematological Study of Thrombocytosis in Children

**DOI:** 10.1155/2014/389257

**Published:** 2014-01-29

**Authors:** Nathiya Subramaniam, Suneel Mundkur, Pushpa Kini, Nalini Bhaskaranand, Shrikiran Aroor

**Affiliations:** Department of Paediatrics, Kasturba Medical College, Manipal University, Manipal, Karnataka 576104, India

## Abstract

*Introduction*. Primary thrombocytosis is very rare in children; reactive thrombocytosis is frequently observed in children with infections, anemia, and many other causes. *Aims and Objectives*. To identify the etiology of thrombocytosis in children and to analyze platelet indices (MPV, PDW, and PCT) in children with thrombocytosis. *Study Design*. A prospective observational study. *Material and Methods.* A total of 1000 patients with thrombocytosis (platelet > 400 × 10^9^/L) were studied over a period of 2 years. Platelet distribution width (PDW), mean platelet volume (MPV), and plateletcrit (PCT) were noted. *Results. *Of 1000 patients, 99.8% had secondary thrombocytosis and only two children had primary thrombocytosis (chronic myeloid leukemia and acute myelogenous leukemia, M7). The majority of the children belonged to the age group of 1month to 2 years (39.7%) and male to female ratio was 1.6 : 1. Infection with anemia (48.3%) was the most common cause of secondary thrombocytosis followed by iron deficiency alone (17.2%) and infection alone (16.2%). Respiratory infection (28.3%) was the predominant infectious cause observed. Thrombocytosis was commonly associated with IDA among all causes of anemia and severity of thrombocytosis increased with severity of anemia (*P* = 0.021). With increasing platelet count, there was a decrease in MPV (<0.001). Platelet count and mean PDW among children with infection and anemia were significantly higher than those among children with infection alone and anemia alone. None were observed to have thromboembolic manifestations. *Conclusions*. Primary thrombocytosis is extremely rare in children than secondary thrombocytosis. Infections in association with anemia are most commonly associated with reactive thrombocytosis and severity of thrombocytosis increases with severity of anemia.

## 1. Introduction

Thrombocytosis refers to platelet count >4,00,000/*μ*L in the peripheral blood [1]. With the widespread use of electronic cell counters and the subsequent availability of a platelet count as part of a routine blood count, thrombocytosis is more often observed as an unexpected finding. Thus, an elevated platelet count has become an important clinical problem for differential diagnosis of various pathological and physiological processes [2, 3].

Thrombocytosis is classified according to its origin into primary and secondary forms. Primary (clonal) thrombocytosis is a myeloproliferative disorder, caused by abnormal and uncontrolled expansion of haematopoietic cells, which is likely to be complicated by thromboembolism [2]. Secondary (or reactive) thrombocytosis is due to a variety of underlying conditions like infection, inflammation, iron deficiency, tissue damage, hemolysis, severe exercise, malignancy, hyposplenism, and other causes of an acute phase response [4]. It is sometimes difficult to differentiate between both categories on clinical ground alone.

Once thrombocytosis is identified and confirmed by peripheral smear, the diagnostic evaluation turns to determine whether the process is reactive or clonal in nature. An important initial step in this is familiarity with the underlying causes of thrombocytosis.

There have been few prospective studies on the clinical circumstances surrounding paediatric thrombocytosis and evaluation of platelet indices (mean platelet volume, platelet distribution width, and plateletcrit) in presence of thrombocytosis.

## 2. Materials and Methods

This prospective observational study was carried out in the Department of Paediatrics, Kasturba Hospital, Manipal, from December 2011 to June 2013. All children aged from 1 month to 18 years admitted in Paediatrics Department of Kasturba Hospital in whom thrombocytosis (platelet > 400 × 10^9^/*μ*L) was identified during routine blood investigations were included in the study. Repeat samples with thrombocytosis were excluded in the study. The approval of the ethical committee was taken prior to the commencement of the study.

Two mL of EDTA blood was collected through a clean venipuncture from children admitted in Paediatrics Department for whom blood investigations were planned and samples were sent immediately to clinical laboratory. The duration from the time of venipuncture to time of using the autoanalyzer was observed to be less than 1 hour. Children in whom thrombocytosis was identified were included in the study. Presenting symptoms and signs with which those children were admitted to the hospital were recorded as per the structured and prevalidated pro forma. Any history of blood transfusion in the previous one month or history of receiving drugs that can cause thrombocytosis (steroid and vincristine) or splenectomy in the past was noted. Hb, WBC counts, red cell indices, PDW, mean platelet volume, and plateletcrit as given by the automated blood cell analyzer (Beckmann Coulter) were noted in all cases with thrombocytosis, along with peripheral smear. Other relevant investigations done to establish the diagnosis were also noted.

Severity of thrombocytosis was graded as follows:

1. mild (400 × 10^3^/*μ*L–700 × 10^3^/*μ*L),
2. moderate (700 × 10^3^/*μ*L–900 × 10^3^/*μ*L),
3. severe (more than 900 × 10^3^/*μ*L).

Normal values of mean platelet volume (MPV) [5], platelet distribution width (PDW) [6], and plateletcrit (PCT) [7] were 7.4 to 10.4 fL, 10–17.9%, and 0.17 to 0.34%, respectively. Based on hemoglobin levels (as per WHO classification) [8] cases were classified into mild, moderate, and severe anemia in children <5 yrs and 5–18 yrs of age group.

Etiology of thrombocytosis was identified by analyzing clinical details and evaluating laboratory parameters. The data was analyzed using the software Statistical Package for the Social Sciences (SPSS v16.0). For the comparison of means and proportions between different groups, unpaired Student's *t*-test and Chi-square test were used, respectively. For correlation studies, Pearson correlation coefficient (*r*) was used.

## 3. Results

A total of 1000 patients aged from 1 month to 18 years were studied from December 2011 to June 2013. Among those children, 39.7% (*n* = 397) belonged to the age group of 1 month to 2 years, 32% (*n* = 320) were between 2 and 5 yrs, and children aged more than 5 years accounted for 28.3% (*n* = 283) of cases. Thrombocytosis was observed to be more common among boys (61.2%) than girls (38.8%) in all age groups (*P* value = 0.05) with overall male to female ratio of 1.6 : 1.

Majority of children (*n* = 889) in the study group had mild thrombocytosis (88.9%). Moderate (*n* = 75) and severe (*n* = 36) thrombocytosis were seen in 7.5% and 3.5% of children, respectively (Table 1).

In the present study, fever (50.1%) was the predominant presenting symptom followed by respiratory symptoms like cough (35%) and respiratory distress (8.8%). Pallor (30%) was the most commonly observed sign followed by hepatomegaly (18.5%), splenomegaly (6.4%), and hepatosplenomegaly (13.5%).

Fever was most commonly associated with severe thrombocytosis than mild thrombocytosis (*P* value: 0.002).

Table 2 shows etiology of thrombocytosis. Among 1000 children, primary thrombocytosis was found in only two cases (one CML and one AML-M7); the remaining children had secondary thrombocytosis.

It was observed that infection with anemia (48.3%) was most commonly associated with secondary thrombocytosis. In 162 children, thrombocytosis was secondary to intercurrent infection alone and anemia (without infection) was the etiological cause in 172 patients. Among infections, respiratory tract infections (*n* = 283) were most commonly associated with thrombocytosis (28.3%). Other infections were urinary tract, gastrointestinal, hepatobiliary and central nervous system infections.

Iron deficiency was most commonly associated with thrombocytosis among all kinds of anemia. It was observed that the more the severity of anemia was, the more severe the thrombocytosis was and it was statistically significant.

Thrombocytosis was found in 21 patients with hemolytic anemia, of whom 14 patients were suffering from beta thalassemia major (12 out of them were splenectomised), 2 from hereditary spherocytosis (1 post splenectomy), and 3 from immune hemolytic anemia (1 after splenectomy). Other hemolytic kinds of anemia included congenital dyserythropoietic anemia (1) and mixed hemoglobinopathy (1).

Thrombocytosis following acute hemorrhage was seen in 9 children, among whom 5 were hemophiliac, 2 children had Von Willebrand disease (VWD), 2 children with hypofibrinogenemia, and one child had subdural hemorrhage following trauma. Other hematological conditions associated with thrombocytosis include acute lymphocytic leukemia (1), hodgkin lymphoma (1), and sinus histiocytosis (1). All the 3 children had thrombocytosis at presentation.

Rebound thrombocytosis (after recovery from thrombocytopenia) occurred in 19 patients and Kawasaki disease was diagnosed in 7 children. Among the study population, in 23 patients, an offending medication that could cause thrombocytosis was given in the preceding one week. Out of these 23 patients, 16 children were on steroids for various ailments. One child with chronic idiopathic thrombocytopenic purpura was found to have thrombocytosis on a single occasion during steroid therapy. Eight children with acute lymphoblastic leukemia were detected to have thrombocytosis during chemotherapy. Among them, seven children had received vincristine in the preceding week and one child was on prednisolone.

Mean MPV of children with mild thrombocytosis was 7.51 fL, moderate thrombocytosis was 7.2 fL, and of severe thrombocytosis was 7.17 fL. With increasing platelet count, there was a decrease in mean platelet volume and there was a weak significant negative correlation (*P* < 0.001) (Table 3).

Mean PDW of children with mild thrombocytosis was 16.55% and those with moderate and severe thrombocytosis were 16.75% and 16.56%, respectively. There was no statistical correlation between severity of thrombocytosis and PDW (Table 3).

Platelet count and mean PDW among children with infection and anemia were significantly higher than those among children with infection alone and anemia alone.

During the study period, none of the children were observed to have thromboembolic manifestations.

## 4. Discussion

Thrombocytosis is common in childhood. However it did not receive much attention in the paediatric literature probably because thrombocytosis is rarely a cause of symptoms and is mainly secondary to other underlying conditions. Reactive thrombocytosis has an estimated incidence of 6–15% among hospitalised children [9] and in the present study 99.8% of the study group had reactive thrombocytosis. Among the two children with primary thrombocytosis, one child was diagnosed with chronic myeloid leukemia (CML) and the other child with acute myeloid leukemia (M7). Both the children had mild thrombocytosis at presentation and had no thrombotic symptoms. In study in 2010 [10], among 250 cases, 3 had primary thrombocytosis and in another study [11], among 663 patients, no case of primary thrombocytosis was observed.

The incidence of reactive thrombocytosis in childhood shows an age-dependent pattern. The highest incidence has been found in infants aged up to 24 months as in the present study. After the age of 2 years, the incidence gradually decreases [12]. In a study [10] thrombocytosis was found to be more common in infants and children aged less than 2 yrs of age (60%). The higher susceptibility for thrombocytosis during the neonatal period may result from various physiological phenomena: a high Tpo gene expression in the bone marrow during the ontogeny of medullary haematopoiesis [13], higher circulating Tpo concentrations in fetuses and neonates than in children and adults [14], and an increased sensitivity of megakaryocytic progenitor cells to Tpo.

Thrombocytosis was observed to be more common among boys (61.2%) than among girls (38.8%) in all age groups. Similar results were observed in two other studies [10, 11], with male preponderance of 64% and 61.1% of cases, respectively. Testosterone has synergistic effect on thrombopoiesis. Clinical and molecular evidence suggests that sex hormones, specifically androgens, mediate platelet count and function [15].

Mild thrombocytosis (88.9%) was seen more commonly than moderate (7.5%) and severe (3.6%) thrombocytosis. Among children with severe thrombocytosis, maximum platelet count observed was 1357 × 10^3^/*μ*L in a child with left pyopneumothorax with anemia. Similar observations were made in a study [10], where mild thrombocytosis was most commonly observed.

Pallor was the most common sign observed among the study population (30%). It was observed that infection with anemia was the most common cause for secondary thrombocytosis (48.3%), intercurrent infections alone in 162 children and anemia alone (without infection) in 172 patients. Among infections, respiratory tract infections (28.3%) were most commonly associated with thrombocytosis.

Seven children were diagnosed to have Kawasaki disease and all of them had thrombocytosis at presentation. Two among them were observed to have severe thrombocytosis. Tpo in conjunction with IL-6 contributes to the thrombocytosis of patients with Kawasaki disease.

Corticosteroids are known to cause transient thrombocytosis, as a result of release of stored platelets from the spleen into the blood circulation [16]. Other drugs that were known to cause thrombocytosis were vincristine and isotretinoin. Among study population, in 23 patients, an offending medication that could cause thrombocytosis was given in the preceding one week.

One child with chronic idiopathic thrombocytopenic purpura was found to have thrombocytosis on a single occasion during steroid therapy. In ITP, megakaryopoiesis is increased in response to immune mediated destruction of platelets. During therapy, at times, platelet overproduction in the phase of decreased or normalized platelet destruction may result in thrombocytosis. Yohannan et al. [11] reported 3 cases with ITP, who developed rebound thrombocytosis following steroid treatment.

Among the noninfectious causes of secondary thrombocytosis, iron deficiency is a common one, since it is the single most common nutritional deficiency worldwide [17]. The fact that thrombocytosis is more frequent in children up to 2 years of age is partly due to the higher incidence of iron deficiency in this age group. In the present study, in children with thrombocytosis, 236 children (23.6%) had mild anemia, 328 (32.8%) children had moderate anemia, and 48 (4.8%) had severe anemia. It was observed that the more the severity of anemia was, the more severe the thrombocytosis was.

In the present study, mean MPV and PDW in the patient with primary thrombocytosis were found to be higher when compared to the mean MPV and PDW of patients with reactive thrombocytosis; however, statistical comparison was not possible.

With increasing platelet count, there was a decrease in mean platelet volume. Platelet count and mean PDW among children with infection and anemia were significantly higher than among children with infection alone and anemia alone.

Reactive thrombocytosis is usually benign and platelet counts normalize rapidly with treatment of underlying etiology without causing any thromboembolic manifestations.

## 5. Conclusions

In paediatric population under most circumstances, thrombocytosis is reactive in nature, most commonly observed among boys under 2 years of age, and primary thrombocytosis is extremely rare. No thromboembolic complications were observed even in cases with severe and extreme thrombocytosis. Infections associated with anemia are the most common cause of reactive thrombocytosis. Among platelet indices, MPV has weak significant negative correlation with platelet count and mean MPV was considerably normal in patients with reactive thrombocytosis. Raised MPV and PDW in a child with persistent thrombocytosis may suggest clonal proliferation and needs further evaluation.

## Figures and Tables

**Table 1:** Distribution of study population based on severity of thrombocytosis.

Severity of thrombocytosis	Number of cases (*n* = 1000)	Percent
Mild (400–700 × 10^3^/µL)	889	88.9 %
Moderate (700–900 × 10^3^/µL)	75	7.5 %
Severe (>900 × 10^3^/µL)	36	3.6 %

**Table 2:** Etiology of thrombocytosis.

	*n* = 1000	Percent
(I) Primary thrombocytosis (*n* = 2)		
(a) CML	1	0.1%
(b) AML-M7	1	0.1%
(II) Secondary (reactive) thrombocytosis (*n* = 998)		
(a) Infection and anemia	483	48.3%
(b) Infection alone	162	16.2%
(c) Iron deficiency alone	172	17.2%
(d) Hematological causes	35	3.5%
(e) Medications	23	2.3%
(f) Rebound	19	1.9%
(g) Kawasaki disease	7	0.7%
(h) Idiopathic	97	9.7%

**Table 3:** Correlation of platelet indices (MPV, PDW) with severity of thrombocytosis.

	MPV (fL)		PDW (%)	
	Mean	SD	Mean	SD
Mild (*n* = 889)	7.51	0.89	16.55	0.75
Moderate (*n* = 75)	7.20	0.81	16.75	0.78
Severe (*n* = 36)	7.17	0.71	16.56	0.67
Correlation coefficient	−0.153		0.011	
*P* value	<0.001		0.736	
